# A female with five chambers

**DOI:** 10.1007/s12471-019-01339-3

**Published:** 2019-10-25

**Authors:** K. K. Sahu, A. Doshi, A. K. Mishra, M. Kranis

**Affiliations:** 1 Department of Internal Medicine, Saint Vincent Hospital, 123 Summer Street, 01608 Worcester, MA United States; 2 Department of Cardiovascular diseases, Saint Vincent Hospital, 123 Summer Street, 01608 Worcester, MA United States

## Answer

**Fig. 1:**
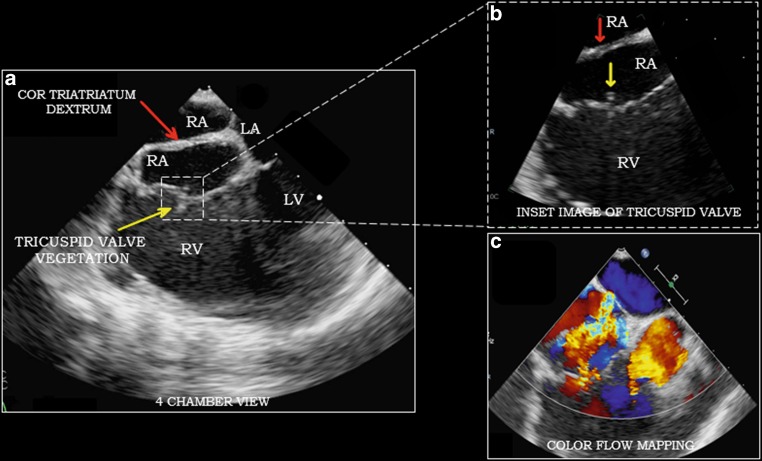
**a**, **b** 2D transoesophageal echocardiography, apical mid-oesophageal four-chamber view of the heart showing presence of a membrane separating the right atrium into two parts (*red arrow*) and vegetation over tricuspid valve (*yellow arrow*); **c** Colour flow Doppler study showing regurgitation at tricuspid valve and back flow of blood through the incomplete septum of cor triatriatum dexter. (*LA* left atrium,* RA* right atrium,* RV* right ventricle,* LV* left ventricle)

This case depicts the classical sequela of intravenous drug abuse in the form of right-sided valve endocarditis. Transoesophageal echocardiography (TEE) shows a vegetation of 0.8 cm on the posterior tricuspid leaflet and severe tricuspid regurgitation with jet velocity of 34 mm Hg (Fig. 1). Also, there was an incidental detection of prominent eustachian valve extending all towards the interatrial septum in right atrium consistent with diagnosis of cor triatriatum dexter (Fig. 1; Video loop 1 and 2). Blood cultures were positive for methicillin-susceptible S. aureus for which treatment was initiated with intravenous cefazolin.

Cor triatriatum is a rare congenital cardiac abnormality in which either the right (dexter) or the left (sinister) atrium gets divided into two compartments resulting in a tri-atrial heart. The sinister variant is more common than the dexter.

Cor triatriatum dexter is the result of persistence of right valve of sinus venosus. The septum is formed as a result of fusion of the eustachian and thebesian valve. It has varying presentations from being asymptomatic to florid right-sided heart failure depending upon the degree of partition of the right atrium. Important to note that cor triatriatum dexter is more than just a membrane and has important clinical implications such as its association with dilated right atrium which is a risk for thrombus formation or arrythmia formation [1]. In most cases, cor triatriatum is detected in infancy due to either heart failure, pulmonary oedema or cyanosis that often requires correction [2]. Very rarely it might be detected in adults incidentally as in our case. Awareness of the rare cardiac entities is essential for timely diagnosis and intervention [3, 4, 5].

## Caption Electronic Supplementary Material

**Video loop 1**. Video, 2D transoesophageal echocardiography showing partition of right atrium by membrane and vegetation on tricuspid valve leaflet

**Video loop 2**. 3D Video loop, transoesophageal echocardiography again confirming the findings of cor triatriatum dexter and tricuspid valve vegetation.

## References

[1] Elagizi A, Marvin R, O’Bryan G (2017). Three’s a crowd—an extremely rare case of cor triatriatum dexter. J La State Med Soc.

[2] Hoye DJ, Wilson EC, Fyfe DA (2010). Cor triatriatum dexter: a rare cause of neonatal cyanosis. Anesth Analg.

[3] Dhibar DP, Sahu KK, Varma SC (2016). Intra-cardiac thrombus in antiphospholipid antibody syndrome: an unusual cause of fever of unknown origin with review of literature. J Cardiol Cases.

[4] Gautam A, Jalali GK, Sahu KK (2017). Cardiac myeloid sarcoma: review of literature. J Clin Diagn Res.

[5] Dhibar DP, Sahu KK, Varma SC, Kumari S, Malhotra P, Mishra AK, Vaiphei K, Khanal S, Suri V, Singhal M (2016). Intra-cardiac thrombus in antiphospholipid antibody syndrome: An unusual cause of fever of unknown origin with review of literature. J Cardiol Cases.

